# Orchestrated efforts on host network hijacking: Processes governing virus replication

**DOI:** 10.1080/21505594.2020.1726594

**Published:** 2020-02-12

**Authors:** Xiaofeng Dai, Olivier Hakizimana, Xuanhao Zhang, Aman Chandra Kaushik, Jianying Zhang

**Affiliations:** a The First Affiliated Hospital of Xi’an Jiaotong University, Xi’an, China; b Wuxi School of Medicine, Jiangnan University, Wuxi, China; c School of Life Sciences and Biotechnology, Shanghai JiaoTong University, Shanghai, China; d Henan Academy of Medical and Pharmaceutical Sciences, Zhengzhou University, Zhengzhou, Henan, China; e Department of Biological Sciences, University of Texas at El Paso, EI Paso, TX, USA

**Keywords:** Virus replication, autophagy, programmed cell death, immune response, cell cycle alteration, lipid metabolic reprogramming

## Abstract

With the high pervasiveness of viral diseases, the battle against viruses has never ceased. Here we discuss five cellular processes, namely “autophagy”, “programmed cell death”, “immune response”, “cell cycle alteration”, and “lipid metabolic reprogramming”, that considerably guide viral replication after host infection in an orchestrated manner. On viral infection, “autophagy” and “programmed cell death” are two dynamically synchronized cell survival programs; “immune response” is a cell defense program typically suppressed by viruses; “cell cycle alteration” and “lipid metabolic reprogramming” are two altered cell housekeeping programs tunable in both directions. We emphasize on their functionalities in modulating viral replication, strategies viruses have evolved to tune these processes for their benefit, and how these processes orchestrate and govern cell fate upon viral infection. Understanding how viruses hijack host networks has both academic and industrial values in providing insights toward therapeutic strategy design for viral disease control, offering useful information in applications that aim to use viral vectors to improve human health such as gene therapy, and providing guidelines to maximize viral particle yield for improved vaccine production at a reduced cost.

## Introduction

Viruses impose many health problems, which can cause severe diseases such as acquired immunodeficiency syndrome (AIDs) [1] or prevalent problems such as influenza [2]. It is therefore important to understand cellular alterations caused by viruses during infection, which can be either used to subdue virus replication for clinical disease management or be taken advantage of in the industry toward increased vaccine production against viral diseases.

Viruses undergo immense variations in their epidemiology and pathogenesis, rendering them difficult to control. For example, HIV is featured by extensive genetic diversity due to its high replication rate and error-prone reverse transcriptase [3], and influenza viruses have types A to D where type A can be further divided into 18 distinct hemagglutinin and 11 neuraminidase subtypes [4]. Hemagglutinin (HA) and neuraminidase (NA) are the two glycoproteins of the influenza virus membrane that both recognize sialic acids; while HAs bind to sialic acids on carbohydrate side chains of cell surface glycoproteins and glycolipids during the initiation of virus infection, NAs remove sialic acids from infected cell surfaces to allow the release of newly generated viruses to infect more cells [5]. These viral surface glycoproteins undergo antigenic drift that allows for evasion from preexisting humoral immunity [6]. It is therefore of the guiding importance to decipher the primary cellular changes that occur during virus infection with the hope of establishing universal therapeutic strategies targeting viral diseases. The target could be, for example, a panel of pivotal cell signaling players, each controlling one/multiple primary cell process(es) that affect(s) virus replication and having alternatives to maintain normal cell functionalities, or a region where multiple signaling pathways overlap in part of the larger global network.

Cell-based approaches for vaccine production use animal cells for vaccine manufacturing, which have more elasticity (regarding the diversity of virus strains feasible for production) than approaches utilizing embryonic eggs [7]. Virus entry and replication processes are orchestrated by a complex network of interactions [8–11]. Murray et al. demonstrated the feasibility of creating an enhanced universal cell line by experimenting with a range of viruses replicating in Vero or Hep-2 cells, and found that knocking down host genes such as *CNTD2, COQ9, GCGR, NDUFA9, NEU2, PYCR1, SEC16G, SVOPL, ZFYVE9, ZNF205* could result in 2-fold to over 1000-fold enhanced replication among 12 tested virus strains [12]. Therefore, it could significantly reduce the cost and complexity of the vaccine production if a universal cell line feasible for the rapid replication of multiple viruses was available where apprehending how viruses hijack host networks to survive is a prerequisite.

With the high demand at both clinical and industrial levels, we are motivated to review the cellular pathways influencing viral propagation and how they function together as an integrated whole to support virus mass production. Through comprehensively reviewing literatures on viral replication, we identified five systems of the host, i.e. autophagy, programmed cell death, immune response, cell cycle alteration, and lipid metabolic reprogramming, frequently reported to be modified during viral infection. Each of these five systems dynamically responds to external or internal perturbations, and represents an integrated collection of processes, e.g. “programmed cell death” includes both apoptosis and non-apoptotic cell death such as pyroptosis and necroptosis.

Our efforts to better understand how cellular networks promote virus replication are twofold in that the information is useful for developing antiviral therapies, and may help optimize cell-based systems for vaccine production to target viral diseases. More specifically, the processes that we cover in this review can inform the selection of genes feasible for targeted control of virus infections.

## Propagation processes

### Autophagy

Autophagy is a process where intracellular materials are engulfed by autophagosomes, delivered to lysosomes for degradation, and consequently recycled to maintain cellular homeostasis [13]. Autophagy could be triggered by virus–host interactions during the entry process, infection-induced cellular stresses, and activated cytosolic and endosomal immune sensors [14]. Autophagy plays dual roles on virus infection, i.e. anti-viral clearance and pro-viral replication [15].

Autophagy can be schematically divided into phagophore initiation, elongation, and fusion with lysosomes, which are executed by different autophagy-related (Atg) genes. In mammals, the unc-51 like autophagy activating kinase 1 (ULK1) complex, autophagy-related protein 13 (ATG13), FAK family kinase-interacting protein of 200 kDa (FIP200), and ATG101 are recruited either on cellular membranes at the ER-mitochondrial junction [16] or at the ER-Golgi intermediate compartment [17] during the initiation phase in a manner strictly regulated by nutrient conditions through mTOR (the mammalian target of rapamycin) [18]. The phagophore is produced by sequential recruitment of autophagy factors and membranes, which facilitates the formation of the curved double-membrane sheet that detaches from the membrane where it originates from. The class III phosphatidylinositol 3 kinase (PI3K) complex contains VPS34, Beclin 1 (ATG6), VPS15, ATG14L or UVRAG (UV radiation resistance-associated gene protein), where the regulatory subunits Beclin 1, VPS15, and ATG14L mediate phagophore membrane expansion during the elongation process. The class III PI3K complex is activated by Beclin 1 dissociation, resulting in the recruitment of PE (phosphatidylethanolamine) and LC3 (microtubule-associated protein 1 light chain 3) that promote the conjugation of lipid molecules [19]. Finally, the class III PI3K complex II which consists of VPS34, VPS15, Beclin 1, and UVRAG is activated to induce the GTPase activity of Rab7 (Ras-associated protein-7) that leads to autophagosome fusion with late lysosomes [20].

As a gate for viral infection control, autophagosomes are considered as an integral part of the immune system. They can deliver cytosolic pathogen-associated molecular patterns (PAMPs) to endosomal pattern recognition receptors (PRRs) and major histocompatibility complex (MHC) loading compartments for innate and adaptive immune stimulation, respectively, or directly exert foreign particle clearance roles by degrading virions [21]. For example, Lee et al. demonstrated that plasmacytoid dendritic cells could recognize certain single-stranded RNA viruses via TLR7 upon transport of cytosolic viral replication intermediates into lysosomes through autophagy [22].

**Figure 1.**
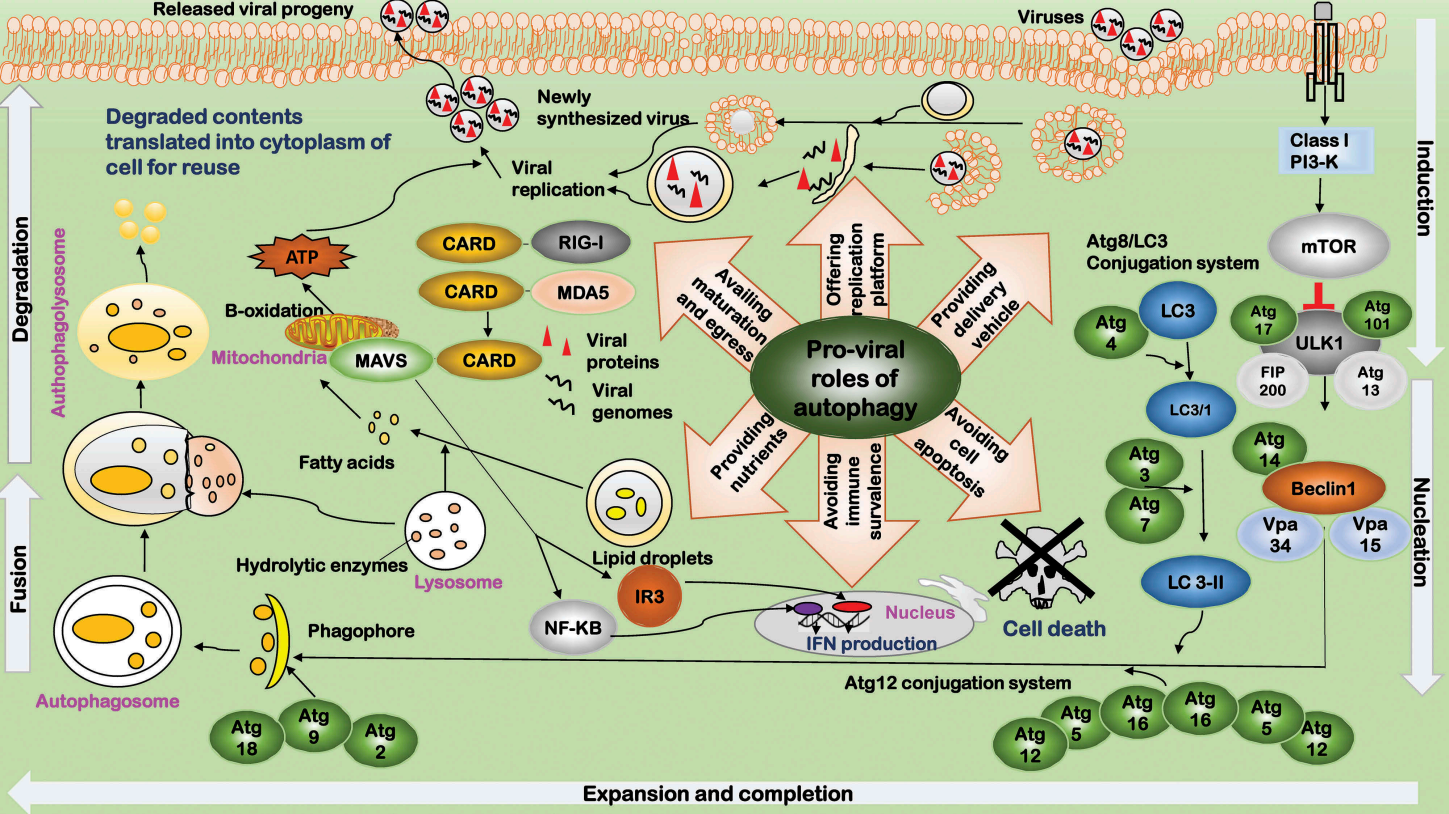
Pro-viral roles of autophagy during virus infection. Autophagy facilitates viral replication in six aspects. 1) Providing delivery vehicle. Viruses hijack host autophagy pathway to deliver viral particles to the replication sites. 2) Offering replication platform. Autophagosomes provide the scaffold and concentrate nutrients for viral replication, and protect the intermediates from immune detection. 3) Providing nutrients. Autophagy provides nutrients for viral replication. 4) Availing maturation and egress. Autophagy machinery facilitates in the late stage of viral lifecycle including capsid maturation, envelopment (if applicable), and egress. 5) Avoiding immune surveillance. Viruses modulate autophagy signaling by inhibiting MHC antigen presentation to avoid immune surveillance and create a favorable environment for viral survival. 6) Avoiding programmed cell death. Viruses have developed strategies to suppress autophagy and avoid programmed cell death. The background depicts key components of the autophagy pathway. The major roles played by autophagy are summarized in the middle, with the relevant components of each role listed close by.

Autophagy can be modulated to favor viral replication (Figure 1) [23]. First, viruses can hijack host autophagy pathway to deliver their particles to the replication sites. For instance, rotavirus can hijack the membrane trafficking pathway of autophagy to transport its viral proteins to the site for genome replication and viral assembly [24], and the same mechanism may also apply to bluetongue virus (BTV) [25]. Second, autophagosomes provide the scaffold and concentrate nutrients for viral replication, and protect the intermediates from immune detection for, mostly, positive-stranded RNA viruses. Examples include poliovirus [26], HIV [27], dengue virus (DENV) [28] and hepatitis C virus (HCV) [29 30,]. Third, autophagy provides nutrients for viral replication. For example, lipid droplets from the degradation of cholesterol in autolysosomes are required for HCV assembly [31]; bluetongue viruses induce autophagy to provide a sustainable cellular environment including, e.g. degraded nutrients and acidic condition to avail their replication [32]. Fourth, autophagy machinery facilitates the late stage of viral lifecycle including capsid maturation, envelopment (if applicable), and virus egress. For instance, proper maturation and envelopment of HIV and HBV involves physical interactions between their glycoproteins and autophagy elements such as LC3 [33]; and autophagosomes help the non-lytic egress of non-enveloped polioviruses through fusing double-membraned vesicles and plasma membrane [34]. Fifth, viruses such as influenza A modulate autophagy signaling by inhibiting MHC antigen presentation, which can prevent viruses from immune surveillance and create a favorable environment for viruses to survive [35]. Lastly, many viruses such as herpes simplex virus-1 (HSV-1) [36], cytomegalovirus (CMV) [37], and Kaposi’s sarcoma herpesvirus (KSHV) [38] have developed strategies to suppress autophagy and avoid degradation.

Host autophagy modulation is not static but dynamically altered by viruses to boost their benefits. Some DNA viruses such as adenovirus [39], simian virus 40 (SV40) [40], human parvovirus B19 (HPVB19) [41], and HBV [42] activate autophagy signaling to enhance viral replication and initiate autophagy-induced cell death as the final step to assist their egress. Many RNA viruses including rotavirus, poliovirus, HCV, influenza virus A, HIV, human T-cell leukemia virus type 1 (HTLV-1) and DENV have been observed to induce autophagy but inhibit autophagosome-lysosome fusion [33,34,42,43].

### Programmed cell death

#### Apoptosis

Apoptosis is a process of programmed cell death required during the development of multicellular organisms for selective cell elimination, and functions as an important mechanism in response to cellular injury [44]. It plays dual roles in viral replication, i.e. protecting cells against viral invasion, or facilitating viral egression. When host cells are infected by viruses, various host cellular molecules involved in cell apoptosis are modulated by viral proteins to enable effective virus replication.

**Figure 2.**
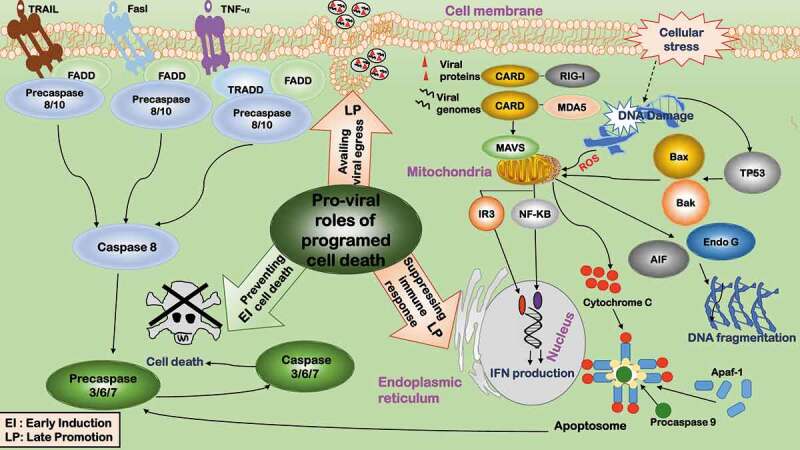
Pro-viral roles of programmed cell death during virus infection. The pro-viral roles related to programmed cell death include: 1) Preventing cell death. Cell death is inhibited during early viral invasion to provide a cellular environment for viruses to replicate. 2) Suppressing immune response. By adopting a tolerogenic type of cell death, programmed cell death paralyzes host’s innate and adaptive defense systems to foster a favorable environment for the progression of viruses and their progenies. 3) Availing viral egress. By subverting host’s immune response, programmed cell death avails viral egress.

Early cell apoptosis after virus infection is a catastrophe for viral survival, and viruses have developed various strategies to block early apoptosis for the maintenance of persistent infection (Figure 2) [45]. Some viruses have evolved certain proteins homologous to BCL2 (a crucial anti-apoptotic process) to inhibit host apoptosis, with E1B 19K proteins of adenovirus, BHRF1 of the Epstein-Barr virus (EBV), and HS5A of HCV being typical examples [46–48]. Some viruses encode viral proteins targeting TP53 given its central roles in growth arrest and apoptosis. While many viral proteins, including adenovirus E1B, the large T antigen of SV40, HBV oncoprotein HBx, IE84 from human CMV, and EBNA5 from EBV, inhibit TP53 activity through direct interactions, E6 of the high-risk human papillomavirus (HPV) serotypes rapidly degrades TP53 via ubiquitin-directed pathway [49,50]. Some viruses encode proteins inhibiting the interleukin-1 converting enzyme family of cysteine proteases, which are pro-apoptotic. Examples here include crmA of cowpox virus, IAP and TP35 of baculoviruses [51 52,]. Many viruses have evolved multiple ways to suppress host apoptosis. Take the influenza A virus for instance, many of its encoding proteins interfere with apoptosis, including neuraminidase that activates BCL2 and the nonstructural protein 1 (NS1) that stimulates the PI3K/AKT pathway [53].

Apoptosis at the late stage of the viral life cycle avails the egress of some viruses by adopting a tolerogenic type of cell death and subverting host’s immune response [54] (Figure 2). Accordingly, many viruses develop strategies to promote apoptosis whenever favorable. Parvovirus infection induces apoptosis via provoking DNA damage and G2/M-phase cell cycle arrest, allowing viral release with less immune response as compared with cell lysis (Figure 2) [55]. Some parvoviruses such as Dependovirus use caspase activity to facilitate replication [56].

Apoptosis triggered in non-infected inflammatory or immune cells could paralyze host’s innate and adaptive defense systems to foster a favorable environment for the progression of viruses and their progenies (Figure 2) [54]. It was observed that T cells isolated from HIV-infected individuals underwent spontaneous apoptosis. Two mechanisms were proposed, i.e. uninfected T cells are capped with CD4 molecules by the HIV-Env proteins from the infected macrophages and thus undergo apoptosis [57], or cell death is triggered via the bystander effect mediated by Fas–FasL interactions [58 59,].

Apoptosis is dynamically reprogrammed to facilitate viral replication. Many viruses initially inhibit cell apoptosis and induce it at a later stage (Figure 2). For instance, rotavirus suppresses apoptosis via viral protein NSP1 to boost virus growth [60], and induces late apoptosis in some cell types via disrupting mitochondrial membrane potential and releasing cytochrome C to minimize the host immune response [61].

Though rare, some viruses induce early cell apoptosis followed by immediate inhibition on cell death using different mechanisms. For example, poliovirus induces cell apoptosis by damaging mitochondria, and early cell death is rescued by anti-apoptotic viral proteins 3A/2B [62]. Also, HSV-1 induces early apoptosis in human epithelial HEp-2 cells by the viral protein IE, and host cells fight against apoptosis later by host proteins [63].

#### Non-apoptotic programmed cell death

Viruses can evoke or suppress several types of non-apoptotic programmed cell death such as pyroptosis and necroptosis. Pyroptosis is characterized by cell lysis and an inflammatory response and may occur as an antiviral immune response to virus infection. For instance, enterovirus 71 (EV71) and coxsackievirus B3 (CVB3) infections activate host pyroptosis, which is accompanied by IL-1β and IL-18 secretion [64]. Necroptosis is marked by rupture of the plasma membrane and release of pro-inflammatory damage-associated molecular patterns (DAMPs), and plays pro-inflammatory roles against virus infection [65]. Viruses can suppress host necroptosis (e.g. disrupting RHIM-RHIM interactions between ZBP1 and RIPK3 or modulating the ubiquitination status of RIPK1 and RIPK3) by secreting necroptosis inhibitors such as ICP6 in HSV-1 [66], ICP10 in HSV-2 [67], and LMP1 in EBV [68].

Relatively little is known about the association between virus infection and other types of non-apoptotic programmed cell death, e.g. oxeiptosis, ferroptosis, MPT (mitochondrial permeability transition)-dependent necrosis, pyronecrosis, parthanatos, PAR polymerase-1-dependent cell death, and NETosis, which await to be explored.

### Immune response

The innate immune response is the first-line host defense against viral invasion, which is rapid and functions toward either infection clearance or process halt until an adaptive response is initiated [69,70]. The innate immune response is interconnected with and fed into the scheme of adaptive response via interferons (IFNs), which belong to cytokines and are required for pathogen elimination [69]. Upon viral infection, the molecular patterns of viruses are detected by various cellular sensors including Toll-like receptors (TLRs) and intracellular helicases such as RIG-I and MDA5, which activate IFN-regulatory factors (IRFs) such as IRF3 to induce the expression of a spectrum of IFN-responsive genes. Typical IFN-responsive gene products include, e.g., protein kinase R (PKR), endoribonuclease L (RNase L), promyelocytic leukemia (PML) nuclear bodies, and cellular restriction factors [71,72]. Viruses have evolved various mechanisms to subvert the host immune response including, e.g. blocking the induction process of IFN response and suppressing IFN response genes.

**Figure 3.**
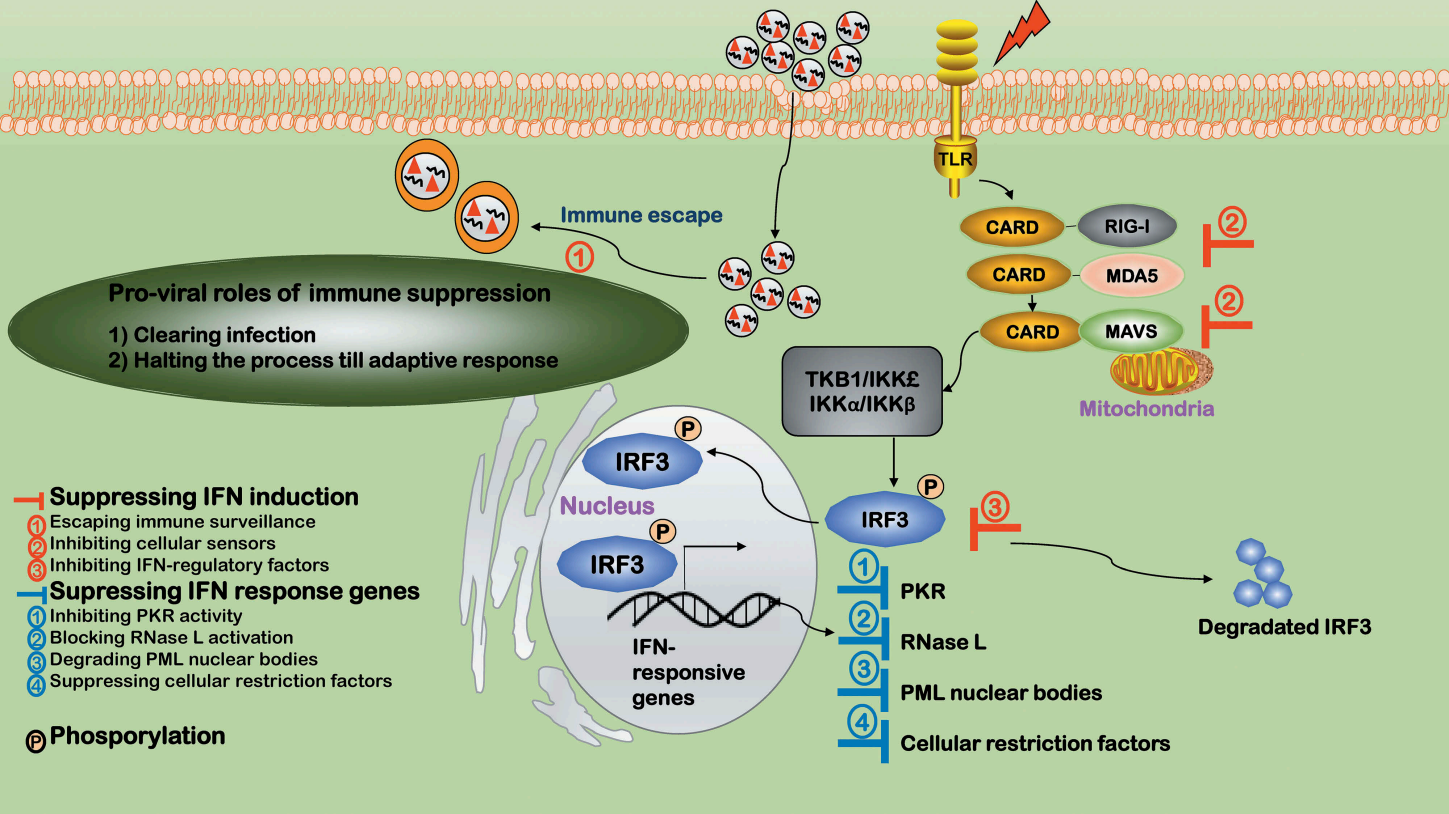
Pro-viral roles of immune suppression during virus infection. Immune suppression favors viral infection in two aspects. 1) Clearing infection. 2) Halting the process till adaptive response. The innate immune response is interconnected with and fed into the scheme of adaptive response via interferons (IFNs). Viruses inhibit immune response by either suppressing IFN induction or IFN response genes. Viruses suppress IFN induction in four ways. ①Escaping immune surveillance. Viruses have evolved various strategies to escape innate immune surveillance. ②Inhibiting cellular sensors. Some viruses target cellular sensors of the immune response such as RIG-I to suppress IFN response. ③Inhibiting IFN-regulatory factors. Some viruses inhibit immune response by targeting IFN-regulatory factors such as IRF3. Viruses suppress IFN response genes, e.g. ①inhibiting PKR activity, ②blocking RNase L activation, ③degrading PML nuclear bodies, and ④suppressing cellular restriction factors.

Mechanisms leading to delayed, weak or no IFN induction are diverse (Figure 3). First, viruses have evolved various strategies to escape innate immune surveillance. For example, HIV-1 modifies PAMPs by altering or hiding its nucleic acids in the viral capsid to mimic the cellular proteins, and randomly mutates its RNA genome to evade host immune recognition [73]; mononegavirale masks its RNA structure by adding a cap to obviate 3ʹppp end exposure from RLR recognition and/or minimizes the production of transcript agonists of RIG-I [74]; West Nile virus evades host anti-viral detection via *de novo* synthesis of 2′-O methylation of the 5ʹ cap of its RNA [75]; flavivirus achieves this by producing the NS5 proteins that perform 2ʹ-O methylation of internal adenosine of viral RNA *in vivo* and host ribosomal RNAs *in vitro* [75]. Second, cellular sensors of immune response such as RIG-I can be targeted by viruses. For instance, HBV produces oncoprotein HBx to prevent the RIG-I induced IFN response and encodes other viral proteins to target adaptor proteins of the RIG-I mediated pathway [76]. Third, viruses inhibit immune response by targeting IFN-regulatory factors such as IRF3. For example, the polymerase of the retrovirus HBV inhibits IRF activation, and rotavirus provides nonstructural protein 1 (NSP1) to degrade IRF3 that induces IFN response on viral infection [60]. Lastly, virus uncoating programs allow capsids to navigate the cell and disassemble in consecutive steps to ensure that genomes are released safely and under precise spatio-temporal control for replication to occur without being detected by the immune system [77].

Many IFN response genes are suppressed during viral invasion (Figure 3). Nearly all classes of viruses encode proteins targeting PKR, e.g. DNA viruses such as vaccinia virus (VACV) [78], adenovirus (ADV) [79], herpes simplex virus (HSV) [80], Epstein-barr virus (EBV) [81], and RNA viruses such as influenza A [82], Ebola virus (EV) [83], HCV [84], rotavirus [85]. Viruses such as HSV-1/2 block RNase L activation by replacing its activators with weak homologues, i.e. the homologue of cellular 2ʹ-5ʹ oligoadenylate [86]. PML nuclear bodies are also IFN-responsive genes typically targeted by viruses including HSV-1, ADV, EBV, HPV, HCV, and rabies virus (RABV). Specifically, ICP0 from HSV-1 degrades PML nuclear bodies [87]; HCV prevents cell apoptosis by co-localizing its core proteins with PML and TP53 [88]; RABV subverts the localization of PML nuclear bodies from the nucleus to cytosol via binding between its P protein and PML [89]. Viruses have also evolved ways to suppress cellular restriction factors, e.g. HIV viral protein VIF degrades APOBECs (incorporable into virions during viral assembly) [90] and VACV E3L protein inhibits ADAR-1 activity (that catalyzes Carbon 6 deamination of adenosine to inosine in double-stranded RNA viruses) [91].

### Cell cycle alteration

The life cycle of a dividing cell can be split into four stages (G1, S, G2, and M) with an additional distinct quiescent stage G0 that occurs outside of the cell cycle. The natural cell cycle consists of a number of checkpoints such as G2/M, G1/S, and G0/G1 to allow cell arrest in response to DNA damage or other environmental changes for the sake of accurate genetic material passaging. Cyclins and cyclin-dependent kinases (CDKs) are the primary components that drive the cell cycle clock by forming an active protein complex. Viruses may subvert the cell cycle in favor of their rapid replication through, primarily, modulating the interactions, phosphorylation, degradation, redistribution, and encoding homologs of cyclins, CDKs, their inhibitors as well as other relevant proteins such as TP53 and checkpoint kinases [92].

#### Cell cycle arrest

Cell cycle arrest halts cell progression at a certain point in the cell cycle where cells are no longer involved in the processes concerning duplication and division. Cell cycle arrest also happens in response to damages such as defects occurred during DNA replication, and the state remains until repair takes place. Viruses may take advantage of cell cycle arrest to modulate the expression of viral or cellular genes critical for the completion of their life cycles given the fluctuating cellular protein levels during cell cycle [93], to reduce the immune response, and to avoid early cell apoptosis (e.g. G0/G1) for the benefit of virus survival [94,95]. Alternatively, viruses may either not be able to pass through one of these checkpoints or not capable of exiting the current phase.

**Figure 4.**
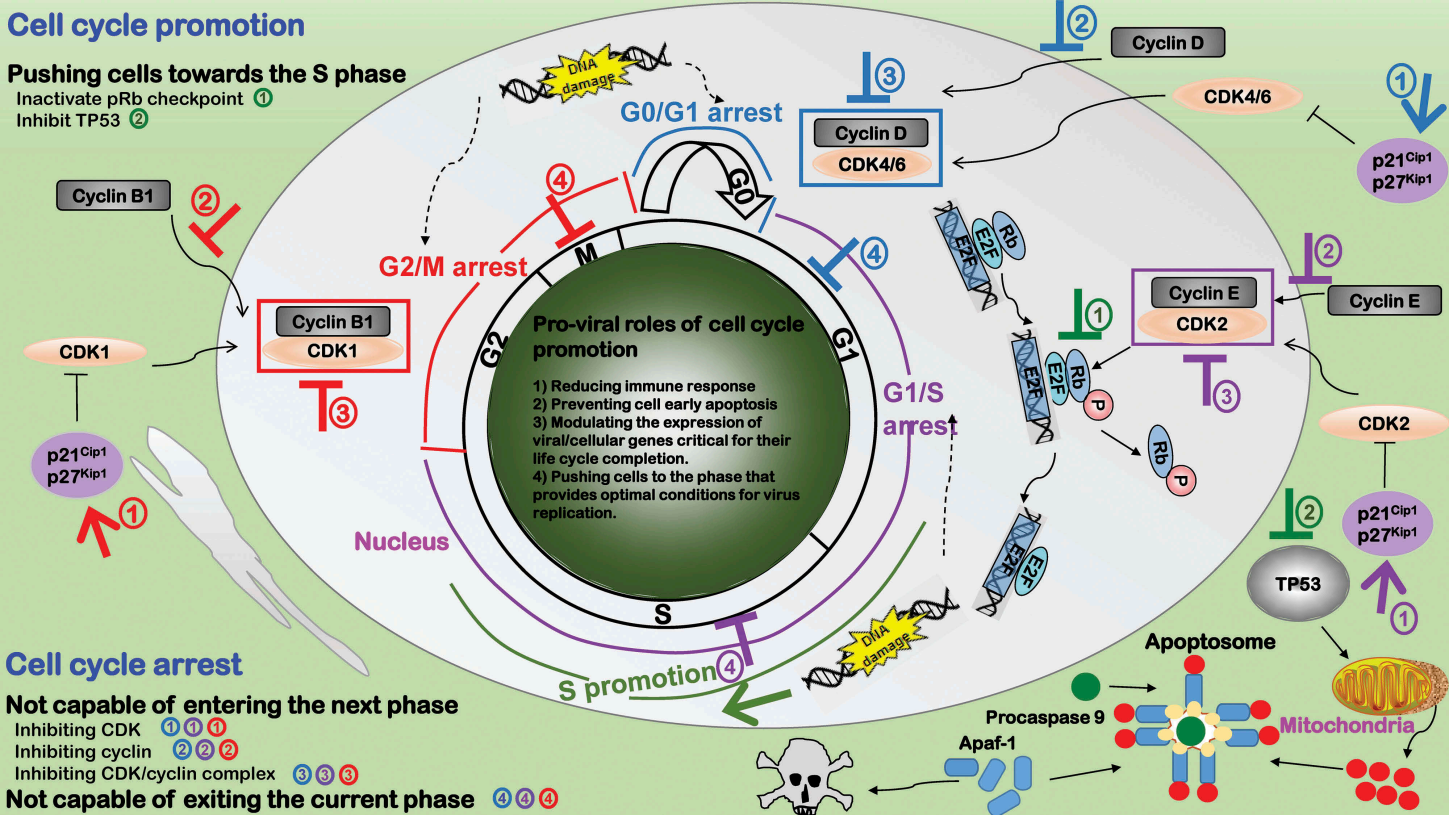
Pro-viral roles of cell cycle alteration during virus infection. Three types of cell cycle arrests exist to favor viral replication in response to DNA damage or other environmental changes for the sake of accurate genetic material passaging, i.e. G2/M (red), G1/S (purple), G0/G1 (blue). Cell cycle arrest plays four pro-viral roles. 1) Reducing immune response. 2) Preventing cell early death. 3) Modulating the expression of viral/cellular genes critical for their life cycle completion. 4) Pushing cells to the phase that provides optimal conditions for virus replication. Cell cycle arrest can be caused by 1) incapable of passing through the checkpoint and entering the next phase or 2) being attracted in or not capable of exiting the current phase. Viruses have developed three strategies to make cells “incapable of passing through the checkpoint”: ①Inhibiting CDK. Some viruses produce proteins to activate CDK inhibitors. ②Inhibiting cyclin. Some viral proteins interact with cyclins. ③Inhibiting CDK/cyclin complex. Some viruses block nuclear transportation or accumulation of CDK-cyclin complexes. Cell cycle promotion (green) typically pushes cells toward the S phase that provides optimal conditions for virus replication through inhibiting pRb and TP53 signalings.

Arrest at the G2/M, G1/S or G0/G1 checkpoint is due to inhibition or delayed activation of CDK1-cyclin B1, CDK2-cyclin E or CDK4/6-cyclin D kinase. Viruses have evolved various strategies to block the formation of these complexes (Figure 4). First, some viruses produce proteins to activate CDK inhibitors such as P21 and P27. For instance, human neurotropic polyomaviruses (JCV) produce agnoprotein to cause G2/M arrest through P21 induction [96]; HBV induces G1/S arrest by modulating the expression of cell cycle-related genes including P21 [97]; influenza A virus generates multiple viral proteins to induce G0/G1 arrest by increasing P21 expression or preventing its proteasome-mediated degradation [94]; the P28 protein from murine coronavirus mouse hepatitis virus (MHV) induces G0/G1 arrest by transcriptionally up-regulating P21 [98]; high P27 level was maintained in the G0/G1 blocked cells after measles virus infection [99]; and the 3B protein from severe acute respiratory syndrome coronavirus (SARS-CoV) could lead to G0/G1 arrest by interfering with cell cycle regulatory genes including CDK inhibitors [100]. Second, some viruses inhibit complex formation by interacting with cyclins. For instance, parvovirus B19 NS1 protein prevents cyclin B nuclear export and thus leads to G2/M arrest [101]; HTLV-I encodes a P30 protein which interacts with cyclin E and leads to cell arrest at G1/S phase [102]; influenza A virus induces G0/G1 arrest by inhibiting cyclin production and stability [94]. Third, some viruses may attract cells in certain states via blocking nuclear transportation or accumulation of CDK-cyclin complexes. For instance, the E4 protein of HPV16 could set cells to G2/M arrest by sequestering the CDK1-cyclin B complex in cytoplasm and preventing it from nuclear entry [103]; HSV-1 and HSV-2 arrest cell cycle in the G1 phase by reducing the level of functional CDK2-cyclin E complexes after infection [104].

During cell cycle state transitions, while most arrested cells are stuck at entry of the next state, some are arrested when exiting the current state (Figure 4). Example strategies that attract cells in the mitotic state include the inhibition on anaphase-promoting complex (APC) by the apoptin protein of chicken anemia virus (CAV) [105], interference with kinetochores by HSV-1 ICP0 protein, and sustained CDK1-cyclin B1 activity by the non-degradable cyclin B analog EC27 protein of baculovirus [106]. Human CMV arrests cells in the S stage by preventing them from genome replication through the IE2 viral protein [107]. HBV triggers hepatocytes exist G0 but stall in G1 through the HBx viral protein that takes advantage of mitochondrial-dependent calcium signaling [108].

One important factor leading to cell cycle arrest that is worth underpinning is DNA damage (Figure 4). DNA damage transfers stress signals via viral proteins or viral genome and causes altered expression of stress-responsive proteins such as TP53 as well as cell cycle regulatory proteins such as CDK inhibitors. For instance, adeno-associated viruses (AAV) could induce the G2/M arrest by activating DNA damage responses [109]; HCVs hijacks the DNA damage response signal, sequesters and relocalizes CDK2 interacting protein (CINP) that is responsible for genomic maintenance from the nucleus to cytoplasm via the NS5B viral protein, and this consequently leads to cell S phase delay [110]; and Epstein-Barr viruses induce TP53-mediated G0/G1 arrest in response to DNA damage via the bZIP transcription factor Zta [111].

#### Cell cycle promotion

The S phase of cell cycle provides optimal conditions required for virus replication as DNA synthesis takes place in the S phase and virus DNA replication depends on the host DNA replication machinery. Promoting host cell cycle from the G0/G1 phase into the S phase is indispensable for some viruses such as polyomavirus which do not encode replication proteins. The pRb and TP53 pathways are critical in the control of transiting cells from one phase to another. Some viruses produce viral proteins to suppress these signalings to promote cell cycle transition from the G0/G1 phase into the S phase. For example, the E1A proteins of AAV inactivate the pRb checkpoint to allow E2F activating genes involved in nucleotide metabolism and DNA replication to prepare materials needed for DNA synthesis in the S phase, and interact with transcriptional modulators of cell cycle to prevent cell cycle arrest and apoptosis by stabilizing TP53 [112]. Similarly, HPVs employ E6, E7 and E5 [113] and polyomavirus takes advantage of large T-Ag and small t-Ag [114] in promoting cells transit toward the S phase.

### Lipid metabolic reprogramming

#### Lipogenesis

Lipogenesis transforms nutrients and metabolic intermediates into fatty acids required for many important cellular functionalities including, e.g. membrane biosynthesis, signal transduction, and energy storage. This process is critical for virus replication from the early stage of entry, through the middle stage of replication and assembly, till the late stage of egress [115].

**Figure 5.**
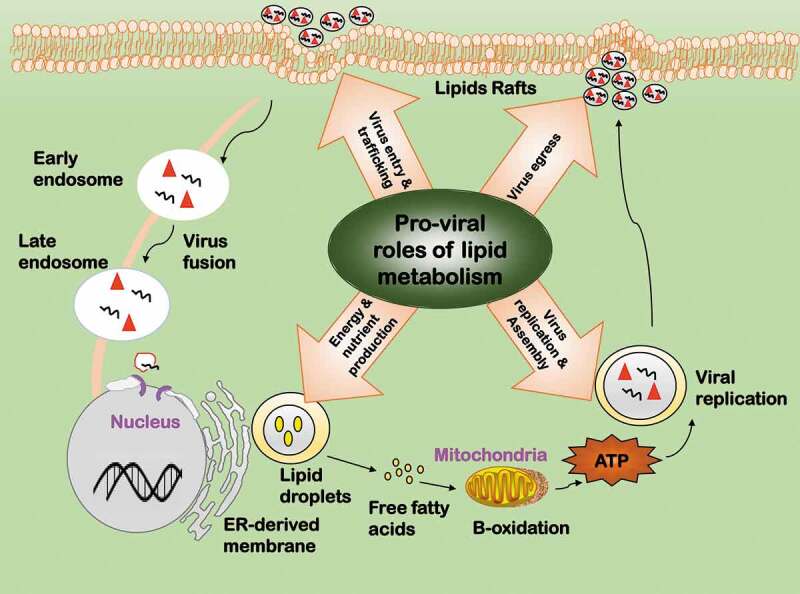
Pro-viral roles of lipid metabolic reprogramming during virus infection. Lipids have four pro-viral roles. 1) Virus entry and trafficking. Lipids can serve as the attachment factor, internalization receptor, or transportation shuttle during viral entry. 2) Virus replication and assembly. Lipids can offer subcellular space for key events to occur in the viral life cycle. 3) Energy and nutrient production. Lipids are required for viral replication as a source of energy and nutrients. 4) Virus egress. Lipids are important components for viral envelopment and play various roles during viral egress.

Viral entry is an early step of the virus life cycle, where lipids can serve as the attachment factor, internalization receptor, or transportation shuttle (Figure 5). The ionic interactions between the virion and cell surface glycosphingolipids aid a number of enveloped and non-enveloped viruses in their entry, as the adhesion of virions to cell membrane permits them to be recognized by the specific internalization receptors [115]. For instance, the infection of subgroup D viruses of adenoviruses occurs after binding to α (2-3)-linked sialic acid (SA), a common carbohydrate component of glycolipids [116], and uses a positive feedback loop between virus uncoating and lipid signaling for efficient membrane penetration [117]. Many RNA viruses and some DNA viruses use lipids such as low-density lipoprotein receptors (LDLR), phospholipids, and gangliosides for entry [118]. For example, glycosphingolipids are indispensable during the entry of parvovirus [119], gangliosides are required for the entry of SV40 [120], LDLR are used by several positive sense RNA viruses including HCV during infection [121]. In addition, viruses such as the double DNA simian virus 40 (SV40) and positive sense RNA echovirus type 1 (EV1) invade cells via caveolin-mediated endocytosis [122]. As caveolins directly bind and concentrate cholesterols and glycol-sphingolipids as well as lipid-modified signaling molecules such as Src-like kinases, eNOS, H-Ras, and heterotrimeric G proteins [123], caveolin-mediated endocytosis during viral invasion considerably enlarges the pool of viruses employing lipids for infection.

One important role of lipids in viral replication lies in the fact that it contributes to organizing subcellular space for key events to occur in the viral life cycle (Figure 5). The majority of RNA viruses such as HCV, dengue virus (DENV) and rotavirus replicate in cell cytoplasm, and some of their viral proteins co-opt in lipid signaling to establish peculiar niches for viral RNA replication [124–126]. For instance, cytoplasmic lipid droplets function as a scaffold to assemble non-structural viral proteins and replication complexes for infectious HCV particle production, and as a vesicle to incorporate and export virus particles outside the cells [127]. Interactions between the lipid kinase phosphatidylinositol-4 kinase III alpha (PI4KIIIα) and the nonstructural viral protein NS5A plays a crucial role in modulating the morphology of viral replication sites in HCV, where NS5A stimulates PI4KIIIα and PI4KIIIα affects NS5A phosphorylation status [128]. Poliovirus genome replication requires continuous phospholipid synthesis, suggesting physical interactions between viral replication complexes and these newborn membranes [129]. Lipids are also important components for viral envelopment and play various roles during viral egress (Figure 5) [126]. Assembly and budding of some viruses such as influenza virus are coupled with budozone formation, which are cholesterol/sphingolipid enriched plasma membrane rafts generated for viral egress [130]. High similarities between the lipid composition of Cyprinus Herpesvirus 3 (CyHV-3) and host lipid rafts reveal the importance of lipid in the envelopment and egress of CyHV-3 [131]. Interestingly, retroviruses use virological synapse for cell-cell communications during their spread.

Viruses have developed different ways to induce fatty acids synthesis and benefit from the intermediates such as lipid droplets, cholesterols, glycosphingolipids, etc. Two enveloped viruses human CMV and vaccinia virus (VACV) achieve this by taking control of central energy metabolisms such as the TCA cycle [132,133]. The enveloped RNA virus HCV modulates lipid synthesis by altering the expression of metabolic related genes, especially cholesterol biosynthesis genes [134].

#### Lipolysis

Lipolysis provides energy and nutrients for viral replication (Figure 5). For instance, DENV infection leads to an autophagy-dependent processing of lipid droplets and triglycerides for free fatty acid release, where ATP and nutrients are generated for efficient DENV replication [135]. Vaccinia virus utilizes *de novo* fatty acid synthesis for ATP generation, where the intermediate product palmitate undergoes β-oxidation and drives tricarboxylic acid (TCA) cycle [133].

## Interrelation between the five identified processes

**Figure 6.**
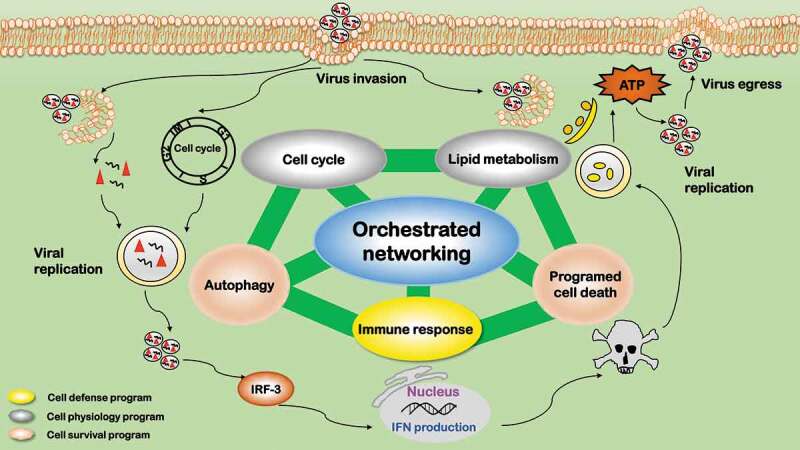
Orchestrated networking among the five influential processes during virus infection. The five influential factors form an orchestrated network governing the fate of viruses after virus infection. Autophagy and programmed cell death are cell survival programs, which are oppositely switched on/off at different stages by viruses to create a favorable environment for virus survival. Immune response is a cell defense program. Cell cycle alteration and lipid metabolic reprogramming are adaptations to cell housekeeping physiologies. Cell defense and physiology adaptations orchestrate and dynamically tilt cells between the two cell survival programs (autophagy and programmed cell death) on virus infection.

Viruses can target and navigate multiple cell networks during and after cell infection. For instance, adenovirus particles exert targeted control of a well-defined neighborhood of networks including endocytosis, autophagy, and microtubule trafficking by coupling several processes to a single protein, protein VI [136]. The five processes we reviewed here do not work alone but orchestrate synergistically to rewire cells fate toward a state favorable for virus replication (Figure 6). Autophagy and programmed cell death are cell survival programs; immune response is a cell defense program; cell cycle alteration and lipid metabolic reprogramming are adaptations on cell housekeeping physiologies. There is an established two-way dialogue among these identified processes, which collectively orchestrate virus replication and determine the fates of invading viruses and host cells.

The two cell survival programs, autophagy and programmed cell death (typically apoptosis), are oppositely switched on/off at different stages by viruses to create a favorable environment for virus replication. Autophagy plays vital roles in retarding mitochondria-mediated apoptosis [137] and is typically modulated reversely with apoptosis. Promoting autophagy while suppressing apoptosis during the early stage of virus infection is common for many viruses. This is because that amphisome formation, nutrient, and energy supply are necessary during virus entry, delivery, and replication, and it is important to maintain a viable host cellular environment before the maturation of viral particles [138]. Late stage suppression of autophagy and activation of apoptosis is favorable for successful infection of many viruses. This is to protect viral progenies from immune-related digestion during viral egression, as apoptosis (being a programmed cell death) does not stimulate immune response.

The cell defense program, i.e. immune response, is primarily suppressed during virus infection. It is well known that certified cells for vaccine or gene therapeutic production such as certified Vero and MDCK cells have defects in their immune defense, e.g. low type I IFN secretion. Immunity can be subverted by autophagy primarily through three mechanisms, i.e. removing endogenous inflammasome agonists [139], degrading immune mediators such as IRF3 [140], and inhibiting type I IFN response via inducing mitophagy [141]. Immunity can be prohibited by apoptosis as apoptotic cells induce immunological tolerance [142]. Immunity can be initiated and modulated by lipids given the unique roles of lipid droplets in maintaining cellular mass homeostasis [143].

The two adaptations on cell housekeeping physiology programs, cell cycle alteration and lipid metabolic reprogramming, can be modulated in either way by viruses to create a favorable environment for their replication. Cell cycle arrest at G2/M, G1/S, or G0/G1, or promotion to the S phase is a common feature during the infection of many viruses. The consequences of cell cycle alteration can be very diverse including, e.g. maintaining a replicative state, preventing the passage of damaged DNA to daughter cells, initiating DNA damage repair and/or suppressing immune response. Such a process is typically associated with cell survival programs and regulated by pRB and TP53-mediated signalings [144]; Cell cycle arrest may lead to less immune response due to reduced cell production [145]. Lipid metabolic reprogramming avails in almost all stages of virus infection, i.e. from initial entry, till replication and assembly, to progeny egress. Lipid droplet, as an energy source, could be produced via cell survival programs to fuel viral production. Autophagy can regulate lipogenesis through modulating the degradation of triglycerides accumulated in cytosolic lipid droplets. Released fatty acids are imported to mitochondria where they undergo β-oxidation to produce ATP for viral replication [146]. Apoptosis is typically accompanied with rapid accumulation of cytoplasmic lipid droplets, by diverting fatty acids away from oxidation to *de novo* lipogenesis [147].

Cell survival programs are the outcome of the collective efforts of cell defense and cell physiology adaptations. Immune response stimulates autophagy by PRRs via autophagic adaptors to eradicate intracellular microorganisms [148]. Interferon, the crucial mediators of innate immune response, induces apoptosis of virus-infected cells and cellular resistance to viral infection [149]. Cell cycle arrest can provoke signals to induce apoptosis, where fatty acids such as butyrate are the pivotal molecules attracting cells in the arrested states [150]. Retroviruses, in particular, set cells to transient cell cycle arrest during its amalgamation into the host genome to activate enzymes necessary for DNA repair, and failed recombination lead to apoptosis [151]. Cell cycle arrest and DNA damage can trigger autophagy in response to cytoplasmic signals [152]. Lipids such as short-chain fatty acids (mostly propionate and butyrate) could induce autophagy [153] and/or apoptosis [154], as substantiated in colon cancer cells.

## Clinical and industrial translation of the identified processes

Viruses have evolved various approaches to rewire “autophagy”, “programmed cell death”, “immune response”, “cell cycle alteration”, and “lipid metabolic reprogramming” toward a favorable state for their survival and replication. The clinical and industrial values of these processes are double-sided. On one hand, developing drugs targeting one or several pivotal genes/proteins that foster the five overlapping cellular processes after viral infection (such as protein VI of adenovirus [136]) may enhance and/or create synergistic effect with the current treatment modalities of virus-related diseases. On the other hand, endogenously or exogenously modulating one or multiple processes may increase the yield of viral particles that become vaccines once inactivated. Strategies modulating these identified processes are not restricted to any particular type of viruses given their unanimous presence, which reduces the clinical treatment complexity and industrial developmental cost.

Several critical points need to be elucidated for appropriate understanding of the five identified processes before translating them into the clinical and/or industrial practice. First, these processes primarily affect virus replication, which do not exclude the importance of other elements in a particular application. For instance, cells feasible for vaccine production cannot be tumorigenic or pathogenic at the first hand. Second, the panel of primary processes driving cell fate reprogramming in a particular infection may be some but not all of the five factors and differ among viruses. Third, underpinning these five identified processes on virus survival and replication does not exclude the probability of other processes contributing to virus replication control. Lastly, the identified 5 processes affecting virus replication correspond to 7 out of the 10 cancer hallmarks [155], i.e. the two cell survival programs could be covered by “sustaining proliferative signaling”, “evading growth suppressors”, “resisting cell death” in cancer hallmarks, the immune response program could be matched to “avoiding immune destruction” and “tumor-promoting inflammation” cancer hallmarks, “cell cycle alteration” identified here and the “enabling replicative immortality” cancer hallmark both refer to cell cycle, and “lipid metabolic reprogramming” is part of the “deregulating cellular energetics” cancer hallmark. Therefore, on one hand, therapeutic strategies against cancer and virus diseases may share some similarities and can thus likely be cross-referenced; on the other hand, it is necessary to check the oncogenic potential of cells established for rapid production of multiple viruses. Three out of the 10 cancer hallmarks are uncovered by the five processes, “genome instability & mutation”, “inducing angiogenesis”, and “activating invasion & metastasis”, which should be used for testing cells’ oncogenic potential through assays examing cell line stability and cell migration.
